# Study of Global Transcriptional Changes of *N*-GlcNAc_2_ Proteins-Producing T24 Bladder Carcinoma Cells under Glucose Deprivation

**DOI:** 10.1371/journal.pone.0060397

**Published:** 2013-04-01

**Authors:** Takahiro Isono, Tokuhiro Chano, Hidetoshi Okabe, Masafumi Suzaki

**Affiliations:** 1 Central Reseach Laboratory, Shiga University of Medical Science, Otsu, Shiga, Japan; 2 Departments of Clinical Laboratory Medicine, Shiga University of Medical Science, Otsu, Shiga, Japan; University of Hawaii Cancer Center, United States of America

## Abstract

Increased levels of *N*-linked (β-*N*- acetylglucosamine)_2_ [*N*-GlcNAc_2_]-modified proteins have been recognized to be an effective response to glucose deprivation. In the first step of this study, using a next generation sequencer, we investigated the global transcriptional changes induced by glucose deprivation in a T24 bladder carcinoma cell line, producing *N*-GlcNAc_2_-modified proteins under glucose deprivation. Our transcriptome analysis revealed significant up-regulation of the UDP-GlcNAc biosynthesis pathway and unfolded protein response genes, and down-regulation of G2/M transition-related genes containing mitotic kinases. Our biological analysis confirmed that *N*-GlcNAc_2_-modified proteins were localized with BiP proteins in the ER. G2/M arrest was caused by glucose deprivation in T24 cells. Moreover, the knockdown of unfolded protein response genes induced the expressional recovery of mitotic kinases under glucose deprivation. Taken together, our results suggest *N*-GlcNAc_2_-modified proteins produced under glucose deprivation caused unfolded protein response in the ER, and that this response induced G2/M arrest.

## Introduction

Glucose metabolism supplies the cell with energy and furnishes carbon skeletons for biosynthesis. Fructose-6-phosphate (Fructose-6-P) is a key metabolite in glucose metabolism, where it primarily enters the glycolytic pathway in order to synthesize ATP and to a lesser extent the pentose phosphate pathway and hexosamine biosynthetic pathway (HBP) to generate metabolites. The HBP produces uridine 5′-diphospho-*N*-acetylglucosamine (UDP-GlcNAc) [1], which is required for *N*-glycosylation in the endoplasmic reticulum (ER), *N*-glycan branching in the Golgi apparatus, and *O*-linked β-*N*- acetylglucosamine (*O*-GlcNAc) modification of proteins in the nucleus, cytoplasm and mitochondria. *O*-GlcNAc-modification of proteins is a feature of many cellular responses to the nutritional state of the cell as well as to stress [2]–[4]. However, *O*-GlcNAc modification of proteins is dependent on the cellular concentration of UDP-GlcNAc. Glucose deprivation was found to induce increased expression of proteins that cross-react with an *O*-GlcNAc specific antibody CTD110.6, despite the decreased concentration of UDP-GlcNAc. These paradoxical phenomena were previously reported in HepG2 human hepatocellular carcinoma cells, Neuro-2a mouse neuroblastoma cells, and some cancerous cells [5]–[8]. However, we have demonstrated that *N*-GlcNAc_2_-modified glycoproteins are also induced under glucose deprivation and that they cross-react with the *O*-GlcNAc-specific antibody CTD110.6 [9]. CTD110.6 antibodies can be used to discriminate *N*-GlcNAc_2_-modified glycoproteins from *O*-GlcNAc-modified glycoproteins by their sensitivity to tunicamycin, which inhibits *N*-glycosylation [9]. It is speculated that immature *N*-linked glycoproteins formed as a result of abundant *N*-GlcNAc_2_-modified proteins is a novel pathway activated in response to glucose deprivation for efficient utilization of this carbon source.

Here, we aimed to discover novel pathways that are activated in response to glucose deprivation in order to clarify the pathophysiological roles of *N*-GlcNAc_2_-modified proteins. In the present study, we employed digital transcriptome analysis using massive parallel sequencing in a next generation sequencer, which has been developed for the quantitative analysis of global transcriptomes [10], [11]. The global transcriptomic changes induced by glucose deprivation were investigated in T24 bladder carcinoma cells. We found that glucose deprivation up-regulated UDP-GlcNAc biosynthesis, and accumulation of *N*-GlcNAc_2_-modified proteins caused an unfolded protein response (UPR) in the ER. This response, in turn, induced cell cycle arrest at the G2/M transition, together with other transcriptional changes.

## Materials and Methods

### Cell lines and cell culture conditions

The human bladder cancer cell line, T24 [12] was usually cultured in the high-glucose version of Dulbecco's modified Eagle's medium (DMEM, Life Technologies, Carlsbad, CA), which contained 25 mM glucose and 1 mM sodium pyruvate, supplemented with 10% fetal calf serum (FCS), penicillin (100 U/ml), and streptomycin (100 µg/ml) at 37°C in a humidified 5% CO_2_ atmosphere. In the experimental culture, cells were seeded in high-glucose medium and then treated with or without transfection of small interference RNA (siRNA) on day 1. The culture medium was then replaced on day 2 with fresh high-glucose medium (25 mM glucose) or with glucose deprivation medium, which was depleted of glucose and sodium pyruvate (0 mM glucose, Life Technologies, Carlsbad, CA). Cells on day 3 and 4 were used for some experiments. The treatments with PUGNAc (100 µM), tunicamycin (2 µg/ml), and etoposide (100 µM) were carried out when the medium was replaced on day 2.

### RNA preparation

Total RNA from T24 cells was extracted using acid guanidinium thiocyanate-phenol-chloroform [13]. The total RNA was quantified with Bioanalyzer (Agilent, Santa Clara, CA) following the manufacturer's instructions. RIN (RNA Integrity Number) and A260/A280 ratio of the prepared total RNA were all 10, and over 1.8, respectively.

### Illumina Genome Analyzer sequencing

The library of template molecules for high throughput DNA sequencing was converted from the total RNA using mRNA-Seq Sample Preparation Kit (Illumina, San Diego, CA) following the manufacturer's protocol. The library was quantified with Bioanalyzer (Agilent) following the manufacturer's instruction. Library (7 pM) was subjected to cluster amplification to cluster generation on a Single Read Flow Cell v4 with a cluster generation instrument (Illumina). Sequencing was performed on a Genome Analyzer GAIIx for 37 cycles using Cycle Sequencing v4 regents (Illumina)..

### Sequence data sets

Human genome build 19 (hg19) were downloaded from University of California, Santa Cruz genome browser (http://genome.ucsc.edu/).

### Data analysis

Image analysis and base calling were performed using Off-Line Basecaller Software 1.6 (Illumina). Reads were aligned using ELAND v2 of CASAVA Software 1.7 with the sequence data sets. Transcript coverage for every gene locus was calculated from the total number passing filter reads that mapped, by ELAND-RNA, to exons. These analyses were performed using default parameters. The data were viewed using Genome Studio Software (Illumina). The advanced analysis for detecting significant pathways was performed using Avadis NGS software (version1.3, Strand Scientific Intelligence Inc., San Francisco, CA). The filterings were performed using default parameters. The genes with significantly different expression were identified by the fold change method (Fold change>2), statistically analyzed by Benjamin-Hochberg's FDR (p<0.05), and categorized into particular pathway categories by Find Significant Pathway analysis (p<0.05). All new data has been deposited in DDBJ/EMBL/GenBank under accession number DRA000417 and DRA000634.

### Antibodies

Anti-*O*-GlcNAc (#MMS-248R, CTD110.6) IgM monoclonal antibody was purchased from Covance (Berkeley, CA). Anti-CD98HC (#9160, H-300) and anti-Laminin β-3 (#20775, H-300) antibodies were purchased from Santa Cruz Biotechnology (Santa Cruz, CA). Anti-α-tubulin (#T9026, DM1A) monoclonal antibodies were purchased from Sigma-Aldrich (St. Louis, MO). Anti-BiP (clone 40) and anti-RB1 (G3-245) monoclonal antibodies were purchased from BD Transduction Laboratories (Franklin Lakes, NJ). Anti-Caspase-3 (#9662) antibodies were purchased from Cell Signaling Technology (Beverly, MA). Anti-Histone H3 phospho-S10 (#5178) antibodies were purchased from Abcam (Cambridge, UK). Alexa 488-labeled anti-rabbit IgG antibodies were purchased from Invitrogen. Cy3-labeled anti-mouse IgG antibodies were purchased from GE Healthcare (Amersham, UK).

### Immunofluorescence staining

Cells were cultured in 35 mm glass bottomed dishes (Greiner Bio-One, Kremsmünster, Austria). For the immunofluorescence staining, the media were removed and the cells fixed with 4% paraformaldehyde for 5 minutes and then permeablized with 0.1% Triton X-100 for 5 minutes. The cells were washed twice with phosphate buffer saline (PBS) before being incubated with a primary antibody overnight. Cells were then incubated with a secondary fluorescence-labeled anti-rabbit or anti-mouse IgG for 1 hour. Samples were observed using a Nikon C1si confocal fluorescent microscope (Nikon, Tokyo, Japan).

### Immunoprecipitation

Cells were lysed in Brij lysis buffer containing 20 mM Tris-HCl (pH 7.4), 150 mM NaCl, 1% Brij 98 (Sigma-Aldrich), and a protease inhibitor cocktail (Nakalai Tesque, Kyoto, Japan). The cell lysate was then clarified by centrifugation at 10,000× *g* for 30 minutes at 4°C. The supernatant was incubated with the specific antibody at 4°C for 1 hour. The immunocomplexes were bound to protein-G sepharose (GE Healthcare) for 1 hour at 4°C and washed five times with TBS-T (10 mM Tris-HCl, pH 7.6, 150 mM NaCl, 0.1% Tween20). The proteins bound to the resin were eluted using Laemmli-SDS buffer and then boiled for 5 minutes. After centrifugation at 10,000× *g* for 2 minutes, the supernatants were analyzed by immunoblotting.

### Immunoblotting

Cells were lysed in Laemmli-SDS buffer, subjected to SDS-polyacrylamide gel electrophoresis, and electro-transferred to membrane filters (Immuno-Blot PVDF membranes, Bio-Rad Laboratories, Richmond, CA). The filters were incubated with a primary antibody in TBS-T containing 2% bovine serum albumin (BSA) overnight and incubated for 1 hour in horseradish peroxidase-conjugated anti-mouse, anti-rabbit (GE Healthcare), or anti-mouse IgM (Sigma-Aldrich) diluted 1∶5,000 in TBS-T containing 2% BSA. Immunoreactivity was detected using the ECL system (GE Healthcare) with LAS4000 (Fujifilm, Tokyo, Japan) and quantified with Multi gauge (Fujifilm), using an anti-α-tubulin antibody as an internal control.

### Cell cycle analysis

Treated cells were harvested with the cultured medium and washed in cold PBS before being fixed with 1% buffered formalin and cold 70% ethanol. The cells were permeabilized and incubated with anti-MPM-2 (diluted 1∶500) (FOXM1, 0.T.181; Abcam, Cambridge, UK) overnight, followed by treatment with Alexa 488 goat anti-mouse IgG (H+L) (Life Technologies). The samples were then washed in cold PBS and transferred to tubes containing 0.1% Sytox Red (Life Technologies). Samples were analyzed on a FACS Caliber flow-cytometer (Becton Dickinson, Franklin Lakes, NJ). The cell proportions labeled by MPM-2 and at various phases of the cell cycle were analyzed with the CELLQUEST software (Becton Dickinson). The data were obtained from experiments performed in triplicate. Statistical comparisons were done with an unpaired t-test. The significance level was set at p<0.01.

### Cell Viability

2×10^5^ cells were plated onto 35 mm dishes and cultured at 37°C. For the cell viability assay, cells were stained with 0.1% trypan blue and the percentage of dead and living cells were determined. Apoptosis was quantified using the annexin V-FITC apoptosis kit (MBL) according to the manufacturer's instructions. Briefly, cells were trypsinized, pelleted by centrifugation, and re-suspended in annexin V binding buffer. FITC-conjugated annexin V (1 µl/ml) and propidium iodide (PI, 0.1 µg/ml) were added to cells and incubated for 15 min at room temperature in the dark. Analyses were performed on a FACSCaliber (Becton Dickinson). The data was analyzed with CellQuest software. The treatment with etoposide (100 µM) was carried out when the medium was replaced on day 2, and the sample was used as a positive control for apoptosis.

### siRNA

siRNA targeting human *ATF3* (s1699), *ATF4* (s1704), and *HOXO1* (s5259) RNA duplexes were purchased from Life Technologies. Scrambled control (D-001810-01-05) RNA duplexes were purchased from Dharmacon, Inc. (Lafayette, CO). Cells were transfected with RNA duplexes using Lipofectamine RNAiMAX reagents (Life Technologies) following the manufacturer's protocol.

### Quantitative reverse-transcription–polymerase chain reaction (qRT-PCR)

Quantitative RT-PCR was performed using the LightCycler 480 SYBG Master I Mix and LightCycler 480 System II (Roche Diagnostics, Mannheim, Germany). Gene expression was normalized using the *GAPDH* gene. Primer sequences are provided in Table S1. All quantification analyses were performed in triplicate.

## Results

### Digital transcriptome analysis of T24 cells under glucose deprivation

**Figure 1:**
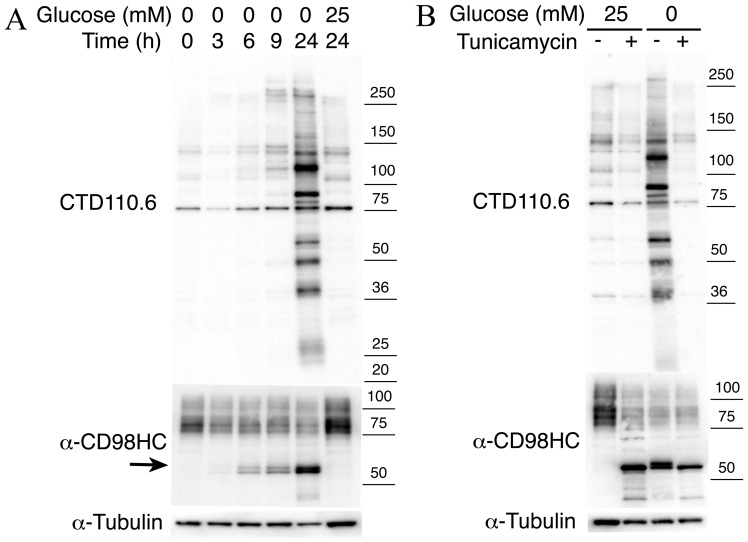
The glycosylation induced under glucose deprivation was not regulated by OGT and *O*-GlcNAcase. **A,** An immunoblot showing CTD110.6 antibody and anti-CD98HC antibody reaction over the time course during incubation of T24 cells in glucose-deprived medium (0 mM glucose). **B,** The effects of tunicamycin treatment on the expression of proteins that reacted with CTD110.6 and anti-CD98HC antibodies under glucose deprivation. An anti-α-tubulin antibody was used as an internal control. Note that *N*-GlcNAc_2_-modified glycoproteins are undetectable after treatment with tunicamycin in medium containing no glucose (0 mM glucose medium). Arrow shows *N*-GlcNAc_2_-modified forms CD98HC.

We used digital transcriptome analysis to study gene expression in T24 cells grown in the absence (0 mM) or presence (25 mM) of glucose. The previous study showed that the expression of *O*-GlcNAc-modified proteins decreased 3 h after glucose deprivation. However, between 6 h and 24 h after glucose deprivation the expression of *N*-GlcNAc_2_-modified proteins increased (Ref 9 and Figure 1). Therefore, we prepared duplicate samples for RNA-seq at 3 h, 6 h, 9 h, and 24 h after glucose deprivation. The control samples with 25 mM glucose were prepared in triplicate. RNA-seq analysis was performed using the ELAND_RNA of CASAVA software and hg19 human genome database as a reference. This analysis resulted in alignments with 14202∼16218 genes from each sample, out of a total of 21407 annotated genes in the database (Table S2). Gene expression was calculated for every gene and given a value corresponding to ‘read per kilobase of exon model’ (RPKM), which is the number of reads aligning to a gene, divided by the length of the gene and the total number of reads [11]. We selected three samples (I, II and III) derived from cells incubated in 25 mM glucose medium. Four samples (0 mM Glucose 6 h-I, II, Glucose 9 h-I and II) were selected as glucose-deprivation samples. We analyzed genes that were differentially expressed in cells incubated in medium lacking glucose (0 mM glucose) and medium containing 25 mM glucose using the ‘Find Significant Pathway’ analysis software package (Avadis NGS software, Table 1). Table 1 shows the most significantly affected pathways i.e., 9 up- and 11 down-regulated pathways (p<0.0001). The up- and down-regulated pathways contained 59 and 84 genes, respectively (each statically two fold different to non-glucose deprived cells) (Table S3).

**Table 1:** The pathways of up- (A) and down-regulated (B) genes in 0 mM glucose condition which were hit by Find significant Pathway analysis.

A			
Pathway	Matched with Technology*	Matched with EntityList†	pValue‡
ATF-2 transcription factor network	49	12	4.55E-10
Glypican pathway	312	18	7.46E-05
HIF-1-alpha transcription factor network	68	9	2.16E-05
Regulation of Insulin-like Growth Factor (IGF) Activity by Insulin-like Growth Factor Binding Proteins (IGFBPs)	12	4	7.59E-05
Regulation of RAC1 activity	116	16	5.78E-09
**UDP-N-acetyl-D-glucosamine biosynthesis II**	**5**	**3**	**8.48E-05**
**Unfolded Protein Response**	**21**	**7**	**1.33E-07**
Validated transcriptional targets of AP1 family members Fra1 and Fra2	100	13	5.19E-07
p53 pathway	156	13	9.85E-06

B			
Pathway	Matched with Technology*	Matched with EntityList†	pValue‡
Androgen-mediated signaling	103	20	3.07E-07
EPHB forward signaling	24	7	9.76E-05
Fatty acid, triacylglycerol, and ketone body metabolism	38	10	1.12E-05
**G2/M Transition**	**10**	**5**	**9.19E-05**
Glypican pathway	312	42	3.67E-08
N-cadherin signaling events	181	28	7.20E-08
Noncanonical Wnt signaling pathway	137	22	1.72E-06
**PLK1 signaling events**	**77**	**14**	**4.42E-05**
Regulation of nuclear beta catenin signaling and target gene transcription	114	19	5.79E-06
Syndecan-4-mediated signaling events	164	25	1.10E-06
Wnt signaling network	144	26	2.19E-08

* : The gene number on the pathway, according to databases.

† : The number of genes, which were statically up and down-regulated in 0 mM glucose condition

‡ : Find signification Pathway analysis (Avadis NGS), Fisher's extract test was performed between EntityList of up and down-regulated genes.

### Genes involved in the UDP-GlcNAc biosynthesis pathway that are up-regulated under conditions of glucose deprivation

**Figure 2:**
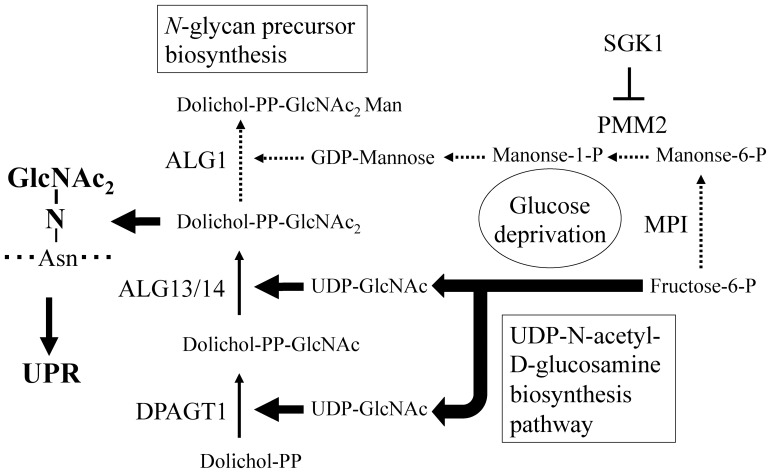
*N*-glycan precursor biosynthesis pathway under glucose deprivation in T24 cells. The solid and dotted arrows indicate activated and repressed metabolite flows, respectively. Note that the production of *N*-GlcNAc_2_-modified proteins under conditions of glucose deprivation is promoted by (i) up-regulation of the UDP-GlcNAc biosynthesis pathway and (ii) suppression in the addition of a mannose residue to dolichol-PP-GlcNAc_2_.

The UDP-GlcNAc biosynthesis pathway was one of the nine pathways up-regulated during glucose deprivation. Specifically, all five genes involved in this pathway showed an increase in expression after 3 h of glucose deprivation, reaching a maximum level of expression after 6–9 h. The expression level of these genes then decreased back to the level observed when cells were incubated in 25 mM glucose medium (Table 2). GFPT1, which was significantly up-regulated during glucose deprivation, is a limiting enzyme in the UDP-GlcNAc biosynthesis pathway. Intriguingly, GFPT1 produces glucosamine-6-phosphate (GlcN-6-P) from Fructose-6-P, a key metabolite in glucose metabolism [14]. The other enzymes that utilize Fructose-6-P as a substrate, which belong to the glycolysis, gluconeogenesis and GDP-mannose synthesis pathways, all displayed decreased levels of expression under glucose deprivation (Table 3). These data suggested that glucose metabolism under glucose deprivation was redirected to the synthesis of UDP-GlcNAc, which is a substrate for *N*-GlcNAc_2_-modification (Figure 2). The elevated level of UDP-GlcNAc biosynthesis will act to inhibit the biosynthesis of GDP-mannose, which is the substrate for adding a mannose residue to dolichol-PP-GlcNAc_2_, through the scramble resource for Fructose-6-P under glucose deprivation (Figure 2). Phosphomannomutase 2 (*PMM2*), a key enzyme of GDP-mannose synthesis, is inhibited by Serum and Glucocorticoid-regulated kinase (Sgk1) [15], [16]. Up-regulated *SGK1* expression (Table S3) would inhibit the biosynthesis of GDP-mannose under glucose deprivation (Figure 2). Taken together, these results suggested that under glucose deprivation the level of *N*-GlcNAc_2_-modified proteins were up-regulated by both the enhanced biosynthesis of UDP-GlcNAc and the inhibition in the biosynthesis of GDP-mannose (Figure 2).

**Table 2:** The changes of digital expression of the genes on UDP-N-acetyl-D-glucosamine biosynthesis pathway after glucose deprivation.

Substrate		Product	Enzyme		Digital Expression: RPKM value (relative)			
			Gene name	25 mM	0 mM_3 h	0 mM_6 h	0 mM_9 h	0 mM_24 h
Fructose-6-P	→	GlcN-6-P	GFPT1*	15.18 (1.00)	26.74 (1.76)	92.26 (6.08)	93.17 (6.14)	53.88 (3.55)
			GFPT2	5.21 (1.00)	10.13 (1.94)	10.59 (2.03)	8.56 (1.64)	4.22 (0.81)
GlcN-6-P	→	GlcNAc-6P	GNPNAT1	17.17 (1.00)	24.70 (1.44)	32.10 (1.87)	24.33 (1.42)	12.85 (0.75)
GlcNAc-6P	↔	GlcNAc-1P	PGM3*	19.93 (1.00)	21.09 (1.06)	66.12 (3.32)	64.49 (3.24)	19.97 (1.00)
GlcNAc-1P	→	UDP-GlcNAc	UAP1*	50.35 (1.00)	165.14 (3.28)	325.21 (6.46)	292.21 (5.80)	61.24 (1.22)

* : Matched genes in Find significant Pathway analysis

**Table 3:** The changes of digital expression of the genes on the output flows of Fructose-6-P after glucose deprivation.

Substrate		Product	Pathway	Enzyme		Digital Expression: RPKM value (relative)			
				Gene name	25 mM	0 mM_3 h	0 mM_6 h	0 mM_9 h	0 mM_24 h
Fructose-6-P	→	GlcN-6-P	UDP-GlcNAc synthesis	GFPT1	15.18 (1.00)	26.74 (1.76)	92.26 (6.08)	93.17 (6.14)	53.88 (3.55)
				GFPT2	5.21 (1.00)	10.13 (1.94)	10.59 (2.03)	8.56 (1.64)	4.22 (0.81)
									
	↔	Manose-6-P	GDP-Mannose synthesis	MPI	41.84 (1.00)	25.16 (0.60)	23.44 (0.56)	33.86 (0.81)	45.34 (1.08)
	↔	Glucose-6-P	Gluconeogenesis	GPI	149.19 (1.00)	111.75 (0.75)	78.29 (0.52)	82.06 (0.55)	100.91 (0.68)
	→	Fructose-1, 6-PP	Glycolysis	PFKL	38.00 (1.00)	27.94 (0.72)	17.96 (0.51)	25.71 (0.68)	28.93 (0.76)
				PFKM	32.57 (1.00)	24.94 (0.77)	17.96 (0.55)	19.04 (0.58)	49.02 (1.50)
				PFKP	104.19 (1.00)	88.89 (0.85)	64.65 (0.06)	59.51 (0.57)	78.72 (0.76)
	↔	Fructose-2, 6-PP	Glycolysis	PFKFB1	0.01 (1.00)	0.02 (2.24)	0.06 (8.21)	0.04 (5.22)	0.06 (8.21)
				PFKFB2	4.35 (1.00)	3.42 (0.79)	3.75 (0.86)	3.07 (0.70)	4.09 (0.94)
				PFKFB3	27.42 (1.00)	10.97 (0.4)	5.955 (0.22)	8.58 (0.31)	14.79 (0.54)
				PFKFB4	16.91 (1.00)	2.65 (0.16)	0.65 (0.04)	1.49 (0.09)	3.47 (0.21)

### *N*-GlcNAc_2_-modified proteins stimulate the unfolded protein response pathway

**Figure 3:**
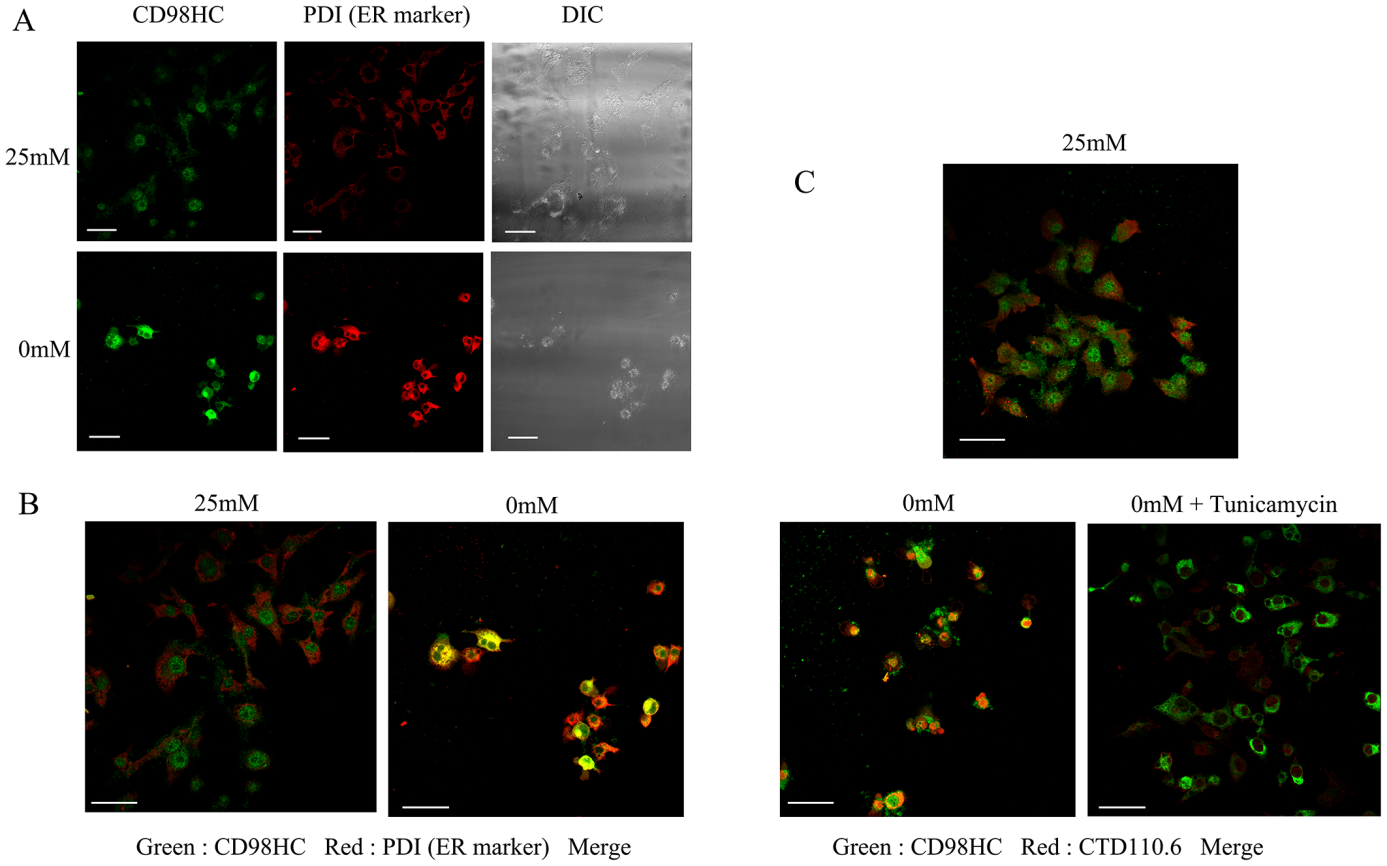
Subcellular localization of CD98HC protein. **A,** T24 cells were cultured in either 25 mM or 0 mM glucose medium for 1day, fixed, and stained with both rabbit polyclonal antibodies for CD98HC and a mouse monoclonal antibody for PDI. The cells were then treated with Alexa 488-labeled anti-rabbit IgG and Cy3-labeled anti-mouse IgG. Fluorescence was observed by confocal fluorescent microscopy. DIC shows photographs obtained using a differential interference contrast microscope. **B,** Enlarged merged photographs. Note that CD98HC and PDI were co-localized under glucose deprivation. **c,** The effects of tunicamycin treatment on the subcellular localization of proteins that reacted with CTD110.6 and anti-CD98HC antibodies under glucose deprivation. T24 cells were cultured either in 25 mM glucose medium, 0 mM glucose-deprived medium, or 0 mM glucose with tunicamycin. The cells were fixed and stained with rabbit polyclonal antibodies for CD98HC and a mouse IgM monoclonal antibody CTD110.6. The cells were then treated with Alexa 488-labeled anti-rabbit and Cy3-labeled anti-mouse antibodies. Merged photographs are shown. Note that CD98HC and CTD110.6 were co-localized under glucose deprivation, but not under glucose deprivation in the presence of tunicamycin. The white scale bars correspond to 50 µm.

**Figure 4:**
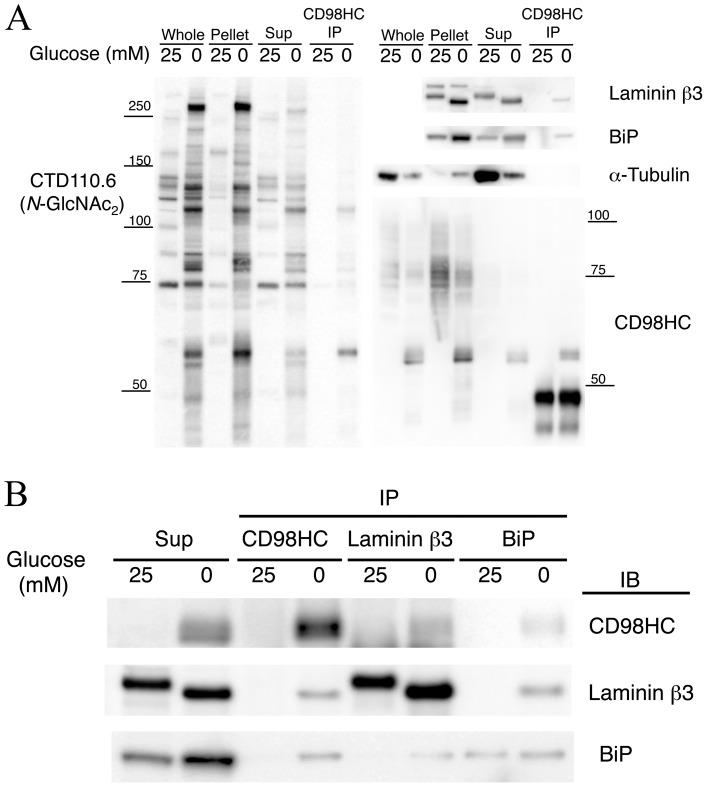
Immunoblot analysis of immunoprecipitated samples. **A,** Immunoblots of immunoprecipitated samples using anti-CD98HC antibody. The left panel shows an immunoblot for CTD110.6. The right panels show immunoblots for laminin β3, BiP, α-tubulin and CD98HC. Note that laminin β3 and BiP are co-precipitated with CD98HC. **B,** Immunoblots showing each immunoprecipitated sample for CD98HC, laminin β3 or BiP. Note that these proteins co-precipitated when the cells were incubated in glucose-free medium (0 mM glucose).

The UPR pathway was among the nine pathways up-regulated when the cells were subjected to glucose deprivation. Indeed, it is reasonable that glucose deprivation would promote UPR. Glucose is required for the glycosylation of proteins. However, when this process is interrupted, unfolded proteins accumulate in the ER thereby initiating the UPR. The *SLC3A2* gene encoding CD98 heavy chain (CD98HC), one of four *N*-GlcNAc_2_-modified proteins [9], was up-regulated. *SLC3A2* belongs to a pathway of validated transcriptional targets of AP1 family members Fra1 and Fra2, when the cells are subjected to glucose deprivation (Table 1 and S3). In fact, under glucose deprivation, *N*-GlcNAc_2_-modified proteins accumulate in the ER where they are involved in UPR. Immunocytochemistry was used to show that CD98HC protein is localized to the cytoplasmic area and is associated with the cytoplasmic membrane when the cells are incubated in 25 mM glucose medium. Under glucose deprivation, however, CD98HC protein did not localize to the cytoplasmic membrane, but instead co-localized to the ER with protein disulfide isomerase (PDI) [17] (Figure 3A, B). CD98HC protein co-localized with the proteins stained by CTD110.6 antibodies. This co-localization is diminished by treatment with tunicamycin (Figure 3C). *N*-GlcNAc_2_-modified proteins, which cross-react with CTD110.6 antibodies, were undetectable after treatment with tunicamycin as described previously [9] (Figure 1B). These changes are consistent with the profile of CD98HC proteins. During glucose deprivation the amount of mature form CD98HC (over 75–100 kDa) decreased coincident with an increase in the amount of *N*-GlcNAc_2_-modified forms (55 kDa; highlighted by arrows) (Figure 1). These results show that *N*-GlcNAc_2_-modified 55 kDa CD98HC proteins accumulated in the ER under glucose deprivation, accompanied by a decrease in the amount of mature 75–100 kDa CD98HC protein localized to the cytoplasmic membrane. Immunoprecipitation analysis confirmed the co-localization of CD98HC proteins and other proteins in the ER (Figure 4). Immunoblot analysis showed that almost all of the CD98HC proteins had a lower molecular mass than the 55 kDa *N*-GlcNAc_2_-modified forms under glucose deprivation as described previously [9] (Figure 1). *N*-GlcNAc_2_-modified CD98HC proteins were co-precipitated with the UPR product, HSPA5 (BiP) [18], under conditions of glucose deprivation (Figure 4). Under the same conditions, *N*-GlcNAc_2_-modified CD98HC proteins were also co-precipitated with laminin β3 protein, another *N*-GlcNAc_2_-modified protein as described previously [9] (Figure 4). The immunoprecipitated fraction with anti-CD98HC antibodies included unidentified *N*-GlcNAc_2_-modified proteins and *N*-GlcNAc_2_-modified CD98HC proteins, which cross-reacted strongly with CTD110.6 antibodies. Moreover, almost all *N*-GlcNAc_2_-modified proteins that were induced under glucose deprivation and cross-reacted with CTD110.6 antibodies, accumulated in the insoluble pellet fraction (Figure 4A). These results suggested that *N*-GlcNAc_2_-modified proteins interacted with one another, accumulated in the ER, and contributed to the UPR pathway.

### Glucose deprivation down-regulates gene expression associated with the G2/M Transition pathway

**Figure 5:**
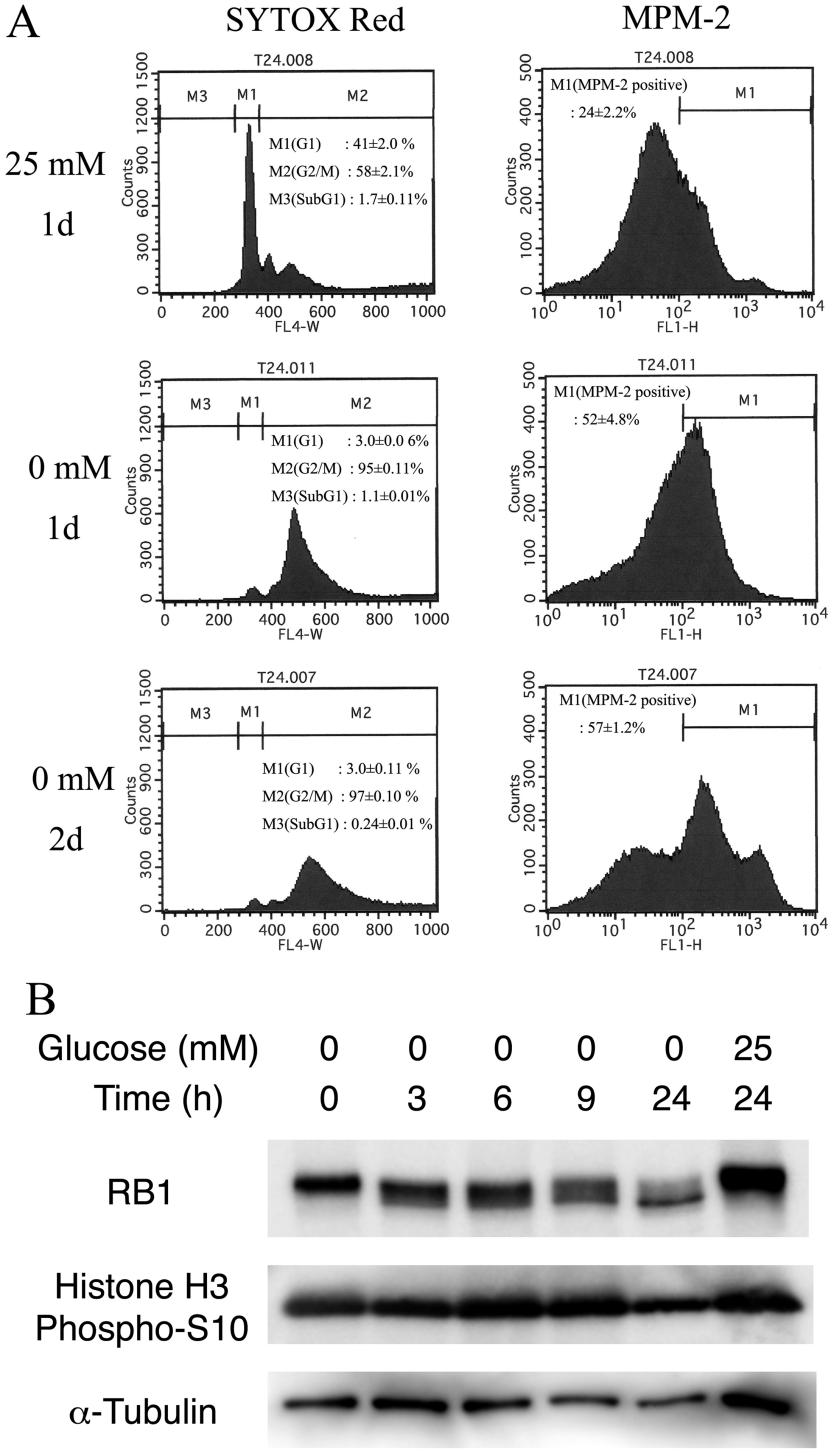
Flow cytometric analysis of T24 cells. **A,** T24 cells were cultured in 25 mM or 0 mM glucose medium for 1–2 days, fixed and stained by SYTOX Red nuclear staining reagent and anti-MPM-2 antibody. Note that 1–2 day glucose deprivation significantly reduced the G1 cell fraction and resulted in the accumulation of G2/M cells stained with anti-MPM-2 antibody. **B,** The panel shows immunoblots for RB1, Histone H3 phospho-S10, and α-tubulin during incubation of T24 cells in glucose-deprived medium (0 mM glucose). Note that the G1/S and G2/M transitions were proceeding but the T24 cells accumulated in early M-phase during glucose deprivation.

The G2/M transition pathway and the pathway of PLK1 signaling events were among the 11 down-regulated pathways under glucose deprivation (Table 1B). These two pathways are related to the cell cycle, especially mitosis. Therefore, we examined the expression of five mitotic kinase genes [19]: *AURKA*, *AYRKB*, *CDK1*, *NEK2*, and *PLK1*, during glucose deprivation (Table 4). All five genes displayed decreased expression 6 h after glucose deprivation and reached the minimal level of expression 24 h after withdrawal of glucose (Table 4). These changes in gene expression were delayed as compared with the other G2/M transition-related genes. Thus, these transcriptional repressions were presumably responsible for inhibiting the G2/M transition in T24 cells during glucose deprivation. We evaluated the cell cycle of T24 cells under glucose deprivation by flow cytometry (Figure 5A). In T24 cells under glucose deprivation for 24 h (1 day), the number of G2/M-phase-cells increased concomitantly with the decreased numbers of G0/G1-phase cells. The cell numbers expressing the early M-phase marker, MPM-2 (FOXM1) [20], also increased during glucose deprivation. To evaluate the cell cycle status, we also performed immunoblots for RB1, phoshorylated in the S-G2/M phases; and for Histone H3 phospho-S10, a marker of early M-phase. Both phosphorylated RB1 and phospho-S10 Histone H3 signals were detected until 9 h. However, signals for all the phosphorylated molecules and α-tubulin decreased significantly at 24 h. These data indicated that the transitions to G1/S and G2/M were proceeding and T24 cells accumulated to early M-phase until 9 h after glucose deprivation (Figure 5B). Finally, the accumulated cells in early M-phase started to die at 24 h after glucose deprivation. Thus we have evidence for a prolonged M-phase of 1–2 days (Figure 5A), together with a loss of protein during the latter part of this period suggesting the initiation of cell death (e.g., mitotic catastrophe).

**Table 4:** The decrease of digital expression of Mitotic kinases (bold) and their related genes (italic) after glucose deprivation.

		Digital Expression: RPKM value (relative)			
Gene name	25 mM	0 mM_3 h	0 mM_6 h	0 mM_9 h	0 mM_24 h
AURKA	59.39 (1.00)	44.07 (0.74)	30.44 (0.51)	23.95 (0.40)	9.38 (0.16)
*TDRD7* *	*12.74 (1.00)*	*7.545 (0.59)*	*4.46 (0.35)*	*5.35 (0.42)*	*8.79 (0.69)*
AURKB	38.49 (1.00)	41.58 (1.08)	22.38 (0.58)	20.66 (0.54)	7.49 (0.19)
*INCENP* *	*26.85 (1.00)*	*13.05 (0.49)*	*9.76 (0.36)*	*13.60 (0.51)*	*5.45 (0.20)*
CDK1	45.01 (1.00)	49.95 (1.11)	35.80 (0.80)	25.41 (0.56)	2.20 (0.05)
*CCNB1*	*119.94 (1.00)*	*115.25 (0.96)*	*92.19 (0.77)*	*63.34 (0.53)*	*16.57 (0.14)*
*CCNB2*	*65.91 (1.00)*	*61.23 (0.93)*	*42.72 (0.65)*	*28.04 (0.43)*	*8.69 (0.13)*
*BUB1B* *	*26.19 (1.00)*	*16.76 (0.64)*	*9.89 (0.38)*	*8.89 (0.34)*	*1.80 (0.07)*
*CDC14B* *	*4.62 (1.00)*	*3.33 (0.72)*	*1.85 (0.40)*	*1.51 (0.33)*	*3.23 (0.70)*
*CDC25B* *	*112.41 (1.00)*	*80.97 (0.72)*	*35.67 (0.32)*	*33.92 (0.30)*	*34.95 (0.31)*
*CDC25C*	*9.03 (1.00)*	*7.86 (0.879*	*5.53 (0.61)*	*5.04 (0.56)*	*1.07 (0.12)*
*RASGRF1* *	*0.33 (1.00)*	*0.16 (0.47)*	*0.10 (0.29)*	*0.11 (0.32)*	*0.64 (1.92)*
*WEE1* *	*15.54 (1.00)*	*9.73 (0.63)*	*7.71 (0.50)*	*6.45 (0.42)*	*3.44 (0.22)*
NEK2	11.06 (1.00)	11.32 (1.02)	8.46 (0.76)	5.92 (0.54)	0.70 (0.06)
*CEP250* *	*16.69 (1.00)*	*10.09 (0.60)*	*4.83 (0.29)*	*6.98 (0.42)*	*11.84 (0.71)*
PLK1	58.63 (1.00)	55.75 (0.95)	31.78 (0.54)	29.23 (0.50)	6.60 (0.11)
*DLGAP5* *	*41.36 (1.00)*	*29.86 80.72)*	*19.29 (0.47)*	*13.88 (0.34)*	*1.51 (0.04)*
*KIF20A*	*39.62 (1.00)*	*29.22 (0.74)*	*16.77 (0.42)*	*11.86 (0.30)*	*0.67 (0.02)*
*NUMA1* *	*57.90 (1.00)*	*31.13 (0.54)*	*18.94 (0.33)*	*25.99 (0.45)*	*35.30 (0.61)*

* : Matched genes in Find significant Pathway analysis

**Figure 6:**
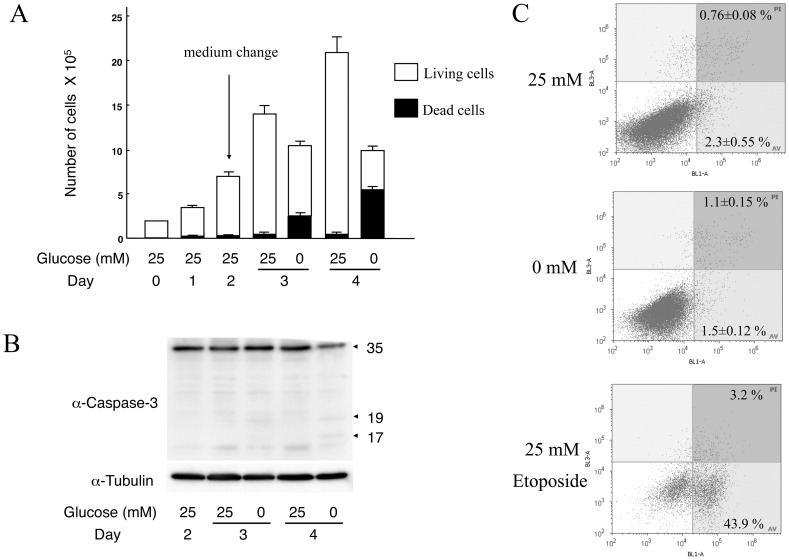
Cell growth and activation of Caspase-3 under glucose deprivation in T24 cells. **A,** The numbers of living and dead cells were counted by the trypan-blue exclusion assay. Error bars represent standard error from three independent experiments. **B,** The panel shows immunoblots for Caspase-3 and α-tubulin. Note that apoptosis of T24 cells is not the primary cause of cell death induced by glucose deprivation. **C,** Flow cytometric evaluation for apoptotic cells during glucose deprivation for 1day, using the annexinV-PI assay. Early and late apoptotic cell populations are shown as ratios (%) of the total cell population. Note that apoptotic cell populations are rare under glucose deprivation, while etoposide significantly induces apoptosis.

In the trypan-blue exclusion assay, glucose deprivation for 1 day stopped cell growth and 21% of the cells were found to be dead (Day 3, 0 mM Glucose in Figure 6A). On day 2 of glucose deprivation, total cell number remained unchanged by comparison with day 1, but the number of dead cells increased to 56% (Day 4, 0 mM Glucose in Figure 6A). In addition, we examined whether apoptosis was induced in T24 cells under glucose deprivation. A subtle cleavage of caspase-3 was observed under glucose deprivation, but this could not fully explain the increase in the number of dead cells (Figure 6B). In flow cytometric analysis, subG1-phase cells were barely detectable at day 2 of glucose deprivation (Figure 5A), despite the increase in the number of dead cells. In another flow cytometric assay of annexinV-PI, glucose deprivation did not induce annexinV-positive apoptotic cell fractions, while etoposide significantly induced such fractions (Figure 6C). Taken together, our findings suggest that cell death induced by glucose deprivation in T24 cells is not primarily the result of apoptosis, but rather the result of prolonged M-phase (e.g., mitotic catastrophe). Indeed, previous studies have shown that mitotic catastrophe is promoted by down-regulation of *CDK1* and *NEK2* genes [21], [22]. Moreover, accumulated and prolonged M-phase cells strongly suggest that glucose deprivation induces cell death such as mitotic catastrophe without apparent apoptosis in T24 cells.

### The knockdown of unfolded protein response-related genes induced expression of mitotic kinase genes under glucose deprivation

**Figure 7:**
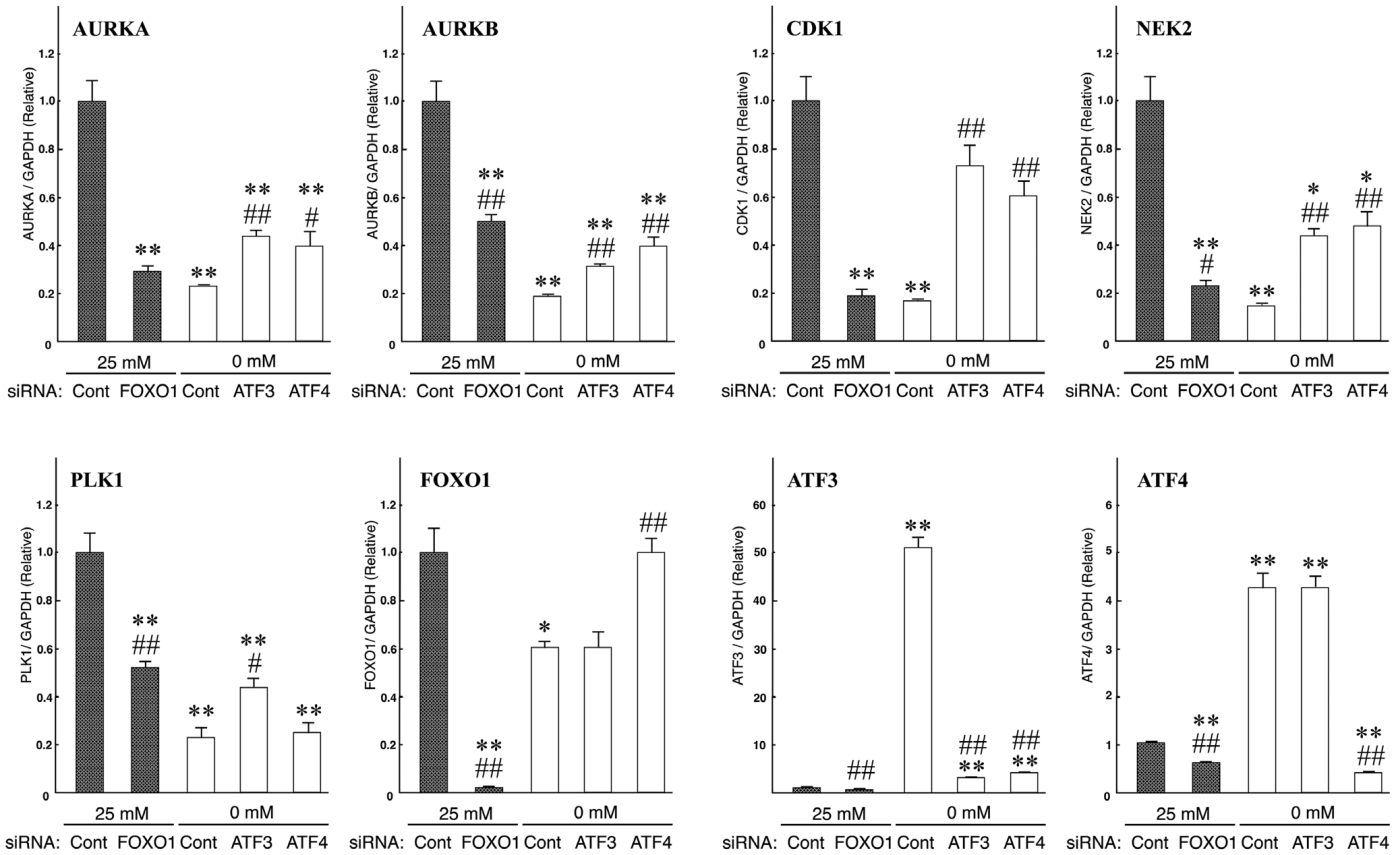
Quantitative RT-PCR of five mitotic kinase genes, under siRNA targeting for ATF3, ATF4 or FOXO1. Quantitative RT-PCR was performed on T24 cells in which siRNA targeted *ATF3*, *ATF4* or *FOXO1*. The cells were either incubated in 25 mM or 0 mM glucose medium for 1 day. Gene expression was normalized using *GAPDH* transcripts. Error bars represent standard errors from three independent experiments. * and **: signify p<0.05 and p<0.01, respectively, against 25 mM glucose with the control-scrambled RNA duplex (siCont) treatment. # and ##: signify p<0.05 and p<0.01, respectively, against 0 mM glucose samples with siCont treatment. Note that the expression of five mitotic kinase genes under glucose deprivation was suppressed. *FOXO1* knockdown inhibited the expression of the five mitotic kinase genes when the cells were incubated in 25 mM glucose medium. Otherwise, knockdown of *ATF3* or *ATF4* allowed the expression of the five mitotic kinase genes to recover when the cells were incubated in medium lacking glucose (0 mM glucose condition).

We examined whether up-regulation of UPR-related genes could contribute to the down-regulation of mitotic kinase genes. *ATF3*, a transcription factor belonging to the UPR pathway, is highly expressed under conditions of glucose deprivation (Table S3A). *ATF4*, another transcription factor belonging to the UPR pathway, positively regulates the transcription of *ATF3* [18], [23]. Therefore, we examined whether mitotic kinase genes were expressed by the knockdown of *ATF3* and *ATF4* genes under glucose deprivation (Figure 7). Quantitative RT-PCR analyses showed that five mitotic kinase genes were significantly down-regulated 24 h after the initiation of glucose deprivation. The result consisted of RNA-seq datasets. The expressional inhibition of *ATF3* gene using siRNA significantly induced the expressional recovery of five mitotic kinase genes (*AURKA*, *AURKB*, *CDK1*, *NEK2* and *PLK1*) under glucose deprivation. The siRNA knockdown of the *ATF4* gene resulted in significant expressional recovery of four mitotic kinase genes under glucose deprivation. In addition, the inhibition of *ATF4* also induced the inhibition of *ATF3* (Figure 7, ATF3 graph). These findings suggested that up-regulation of UPR-related genes (such as ATF4 and 3) could contribute to the down-regulation of mitotic kinase genes.

**Figure 8:**
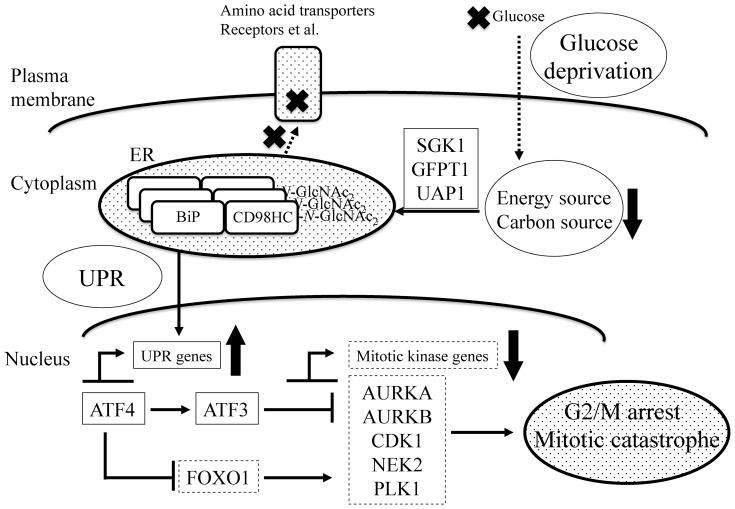
A schematic summary of all the experimental results. The dotted arrows show the transfer of molecules. The thick crosses signify an inhibition in the transfer of molecules. The solid thin arrows indicate activation or repression of targets. The solid thick arrows signify either an increase (up arrow) or decrease (down arrow) in the level of the corresponding molecules. The solid and dotted squares surrounding the gene names indicate up- and down-regulated gene expression, respectively. Note that *N*-GlcNAc_2_-modified proteins accumulated in the ER induce G2/M transition arrest *via* the unfolded protein response (UPR) under conditions of glucose-deprivation.

We screened another key factor that linked up-regulation of UPR-related genes to the down-regulation of mitotic kinase genes. *FOXO1*, a transcription factor belonging to the androgen-mediated signaling pathway, is significantly repressed under conditions of glucose deprivation (Table S3B). It has been reported that *FOXO* family genes induce G0/G1 transition and arrest in the G2/M transition [24]. Under normal (25 mM) glucose conditions, RNAi mediated knockdown of *FOXO1* significantly repressed five mitotic kinases (*AURKA*, *AURKB*, *CDK1*, *NEK2* and *PLK1*). The level of repression was similar to that observed under conditions of glucose deprivation (Figure 7). *ATF4* knockdown under glucose deprivation resulted in a significant recovery in expression of the *FOXO1* gene, while knockdown of *ATF3* did not induce any such affect (Figure 7, FOXO1 graph). These results suggest that *ATF4* up-regulation induced both *ATF3* up-regulation and *FOXO1* down-regulation under conditions of glucose deprivation, and continuously repressed the mitotic kinase genes (Figure 8).

## Discussion

Our digital transcriptome analysis of T24 bladder carcinoma cells under glucose deprivation showed up-regulation of the UDP-GlcNAc biosynthesis pathway and UPR pathway; and down-regulation of the G2/M transition pathway containing mitotic kinases. Moreover, we showed that *N*-GlcNAc_2_-modified proteins interact with one another and accumulate in the ER, which can then stimulate the UPR through binding with BiP. Glucose deprivation also induced G2/M transition arrest and probably mitotic catastrophe. Under glucose deprivation, the up-regulation of UPR induced down-regulation of mitotic kinase genes *via* both repression of the *FOXO1* gene and activation of *ATFs*.

Under glucose deprivation, *N*-GlcNAc_2_-modified proteins are produced by the dysfunction of ALG1, which adds a mannose residue to dolichol-PP-GlcNAc_2_ [9]. Here, we show for the first time that the biosynthesis of UDP-GlcNAc, which is a substrate for *N*-GlcNAc_2_-modification, is promoted by up-regulation of genes involved in the UDP-GlcNAc biosynthesis pathway under glucose deprivation. Otherwise, under glucose deprivation, the biosynthesis of GDP-mannose, the substrate for the addition of a mannose residue to dolichol-PP-GlcNAc_2_, was inhibited by competition for Fructose-6-P with the biosynthesis of UDP-GlcNAc and inhibition of PMM2 activities by SGK1. Thus, during glucose deprivation the production of *N*-GlcNAc_2_-modified proteins was strongly promoted by up-regulation of the UDP-GlcNAc biosynthesis pathway and suppression of addition of a mannose residue to dolichol-PP-GlcNAc_2_ (Figure 2). Under glucose deprivation, the increase in the pool of UDP-GlcNAc may affect the production of *O*-GlcNAc-modified proteins, which uses UDP-GlcNAc as a substrate. However, the production of *O*-GlcNAc-modified proteins decreased 3 h after the initiation of glucose deprivation, and then recovered by the 6 h time point without any significant increase above normal levels of production (as shown in Figure 1). These results are explicable because *O*-GlcNAc- modification is a reversible reaction mediated by *O*-linked β-*N*-acetylglucosamine transferase (OGT) and β-D-*N*-acetylglucosaminase (*O*-GlcNAcase) [2]–[4].

We initially reasoned that *N*-GlcNAc_2_-modified proteins might be transferred and function as mature *N*-linked sugar-modified proteins under glucose deprivation. However, our present transcriptome analysis indicates that *N*-GlcNAc_2_-modified proteins induce the UPR without functioning as mature *N*-linked sugar-modified proteins. A previous study showed that the yeast ts-mutant K57-6C, which was unable to add a mannose residue to dolichol-PP-*N*-GlcNAc_2_ at 37°C, secretes *N*-GlcNAc_2_-modified exoglucanase when the growth temperature was shifted from 25 to 37°C, akin to mature *N*-linked sugar-modified exoglucanase [25]. However, our immunocytochemical analysis showed that *N*-GlcNAc_2_-modified CD98HC protein was localized to the ER rather than the cytoplasmic membrane where the mature forms of the protein are found. In addition, *N*-GlcNAc_2_-modified CD98HC protein aggregated with BiP and other *N*-GlcNAc_2_-modified proteins, such as laminin β3. Aggregates in the ER stimulated the UPR. Under normal conditions, CD98HC protein covalently binds to many amino acid transporters (*SLC7A5*-*11*) to become localized at the cytoplasmic membrane [26]. However, under glucose deprivation the *N*-GlcNAc_2_-modified form of CD98HC was localized in the ER (Figure 1). The other amino acid transporters (*SLC1A5* and *SLC6A19*) contribute as a source of energy and carbon, and are regulated by the hexosamine biosynthetic pathway [27]. During glucose deprivation, *N*-GlcNAc_2_-modification of proteins can deregulate these transporters in a similar way to CD98HC. Finally, cancer cells such as T24 cells, which produce *N*-GlcNAc_2_-modified proteins under glucose deprivation, can enter severe metabolic starvation where neither glucose nor amino acids can be utilized (Refer to Figure 8).

Our digital transcriptome analysis also showed a down-regulation of G2/M transition-related genes. Moreover, our flow cytometric analyses showed that glucose deprivation induced T24 cells to enter G2/M transition arrest and probably mitotic catastrophe. Generally, glucose deprivation triggers AMPK activation of p53, which stabilizes the cell cycle inhibitor p27 to elicit G1 cell cycle arrest [28], but the present study demonstrated that transcriptional suppression of multiple mitotic kinase genes [29] contributes to the G2/M transition arrest. Such a starved condition induces G2/M transition arrest and probable mitotic catastrophe in cancer cells such as T24 cells.

In the present study, during glucose deprivation the UPR could contribute to G2/M transition arrest *via* the transcriptional enhancement of *ATF4* and the subsequent repression of mitotic kinases (refer to Figure 8). The UPR induced the expression of *ATF4*, which in turn induced the expression of the *ATF3* gene. In this study, we performed various siRNA knockdowns combined with qRT-PCR evaluation. Our findings show that the induced expression of *ATF3* under glucose deprivation contributed to the suppression of five mitotic kinase genes. *ATF3* has been shown to be a negative regulator of transcription [30]. Thus, *ATF3* may negatively regulate, either directly or indirectly, the expression of the five mitotic kinase genes. Induced *ATF4* can suppress *FOXO1*. The suppressed *FOXO1* can also reduce the expression of the five mitotic kinases. *FOXO* family genes are known to induce arrest at the G0/G1 transition and G2/M transition [24], which is consistent with our present data. Additionally, the UPR may contribute to mitotic catastrophe, which is promoted *via* down-regulation of the *CDK1* and *NEK2* genes [21], [22]. However, further studies will be needed to clarify the mechanistic association between mitotic catastrophe and the UPR under glucose deprivation.

At present, the role of UPR in cancer is very complex and not yet fully characterized. UPR is activated in various tumors such as breast, lung, gastric and hepatocellular carcinoma [31]–[33]. UPR is beneficial to tumor cells since increases protein folding capacity leading growth advantage. However, in other tumors, UPR down-regulation may allow cancer cells to escape the apoptotic pathway and favor tumorigenesis [34]. Therefore the UPR can trigger pro-survival and pro-apoptotic signals and it is important to understand how modulation of this pathway alters the balance between survival and apoptotic processes and contributes to carcinogenesis in different cell types. In the present study, T24 bladder carcinoma cells showed the biphasic increases of UPR genes ATF3 and ATF4. The first increase occurred at 3 h after glucose deprivation before the production of *N*-GlcNAc_2_-modified proteins, and the second one accompanied with the accumulation of *N*-GlcNAc_2_-modified proteins at 9–24 h after glucose deprivation (Table S3). The former may contribute to pro-survival process, and the latter may do to cell death process. Then, sustained UPR could contribute to inhibit M-phase progression and induce cell death such as mitotic catastrophe rather than apoptosis. In addition, our preliminarily unpublished experiments have indicated that some cancer cells (like T24 cells) producing *N*-GlcNAc_2_-modified proteins under glucose deprivation are arrested at M-phase and subsequently die. Intriguingly, however, other cancer cells under glucose deprivation, which do not produce *N*-GlcNAc_2_-modified proteins, are arrested at G1-phase and survive (data not shown).

The present study demonstrates that in T24 cells the production and subsequent accumulation of *N*-GlcNAc_2_-modified proteins stimulates the UPR in the ER, which in turn induces G2/M transition arrest and possible mitotic catastrophe under glucose deprivation (Figure 8). The present modes of digital transcriptome analyses will provide further useful information on mechanistic aspects of cancer cells under starvation conditions.

## Supporting Information

Table S1 Oligonucleotides used for qRT-PCR.
(PDF)

Table S2 Summary of sample data.
(PDF)

Table S3 Lists of genes, which statically two fold up- and down-changed in most significantly up- (A) and down (B)-regulated pathways (p<0.0001) (Ref. Table 1).
(PDF)
