# Correction: Study of Global Transcriptional Changes of *N*-GlcNAc_2_ Proteins-Producing T24 Bladder Carcinoma Cells under Glucose Deprivation

**DOI:** 10.1371/annotation/053f227a-3847-41e2-a8c4-a5530a282a29

**Published:** 2013-11-12

**Authors:** Takahiro Isono, Tokuhiro Chano, Hidetoshi Okabe, Masafumi Suzaki

In Supplementary Table 1, the last line of the control primer sequence should read GAPDH GGGAGCCAAAAGGGTCATCATC TGGCATGGACTGTGGTCATGAG instead of GAPDH GGGAGCCAAAAGGGTCTCATC TGGCATGGACTGTGGCCGAG. 

Please find the corrected Supplementary Table 1 here: 
