# Supplementary material for: Study of Global Transcriptional Changes of N-GlcNAc2 Proteins-Producing T24 Bladder Carcinoma Cells under Glucose Deprivation
Source: PLoS One. 2013 Apr 1;8(4):e60397. doi: 10.1371/journal.pone.0060397 (PMC3613393; doi:10.1371/journal.pone.0060397)
Supplement: Table S1 — Oligonucleotides used for qRT-PCR. (PDF) [file pone.0060397.s001.pdf]

Table S1. Oligonucleotides used for qRT-PCR

Gene	Forward	Reverse
AURKA	ATTTCAGGACCTGTTAAGCTAC	TCTGATTCTGAACCGGCTTGTG
AURKB	CACTTCACAATTGATGACTTTGAG	CAGGATGTTGGGATGGTGCAG
CDK1	TGGAGTTGTATAAGGGTAGAC	ATAAGCACATCCTGAAGACTGAC
NEK2	CCCTGAAGGAATGCCACAGAC	TGCGATTCATTTGTTCAGGAGAC
PLK1	AGATCAACTTCTTCCAGGATCAC	TGAGACGGTTGCTGGCCGAG
ATF3	TGCCTGTCCCCTCCTGGGTC	TCTTCTTCAGGGGCTACCTCG
ATF4	TCCAACAACAGCAAGGAGGATG	GGGCAAAGAGATCACAAGTGTC
FOXO1	AAGCTCCCAAGTGACTTGGATG	AGTACTTTTAAGTGTAACCTGCTC
GAPDH	GGGAGCCAAAAGGGTCTCATC	TGGCATGGACTGTGGCCGAG
