# Supplementary material for: Study of Global Transcriptional Changes of N-GlcNAc2 Proteins-Producing T24 Bladder Carcinoma Cells under Glucose Deprivation
Source: PLoS One. 2013 Apr 1;8(4):e60397. doi: 10.1371/journal.pone.0060397 (PMC3613393; doi:10.1371/journal.pone.0060397)
Supplement: Table S2 — Summary of sample data. (PDF) [file pone.0060397.s002.pdf]

Table S2. Summary of sample data

Sample Name	Number of Lanes	Avg. Cluster Raw	Avg. % PF Cluster	Avg. % Align PF	Genes
25mM Glucose-I	1	218197	88.06	73.38	14974 /21407
25mM Glucose-II	1	369209	73.01	76.38	15303 /21407
25mM Glucose-III	1	521963	60.59	77.39	15700 /21407
0mM Glucose 3h-I	1	329361	77.22	74.35	15543 /21407
0mM Glucose 3h-II	1	283961	83.44	74.44	15489 /21407
0mM Glucose 6h-I	1	381234	40.23	75.37	14202 /21407
0mM Glucose 6h-II	1	253300	88.01	77.11	15520 /21407
0mM Glucose 9h-I	1	321191	80.78	76.18	15283/21407
0mM Glucose 9h-II	1	483075	62.9	71.95	16218/21407
0mM Glucose 24h-I	1	279560	85.41	75.81	14901/21407
0mM Glucose 24h-II	1	222604	87.83	71.47	14873/21407

Number of Lanes : Number of the lane on the flow cell

Avg. Cluster Raw : Average number of cluster per tile

Avg. % PF Cluster : Average % of clusters passing filter

Avg. % Align PF : Average % of clusters that could be aligned to the genome in clusters passing filter

Genes : Number of genes that could be aligned to the genome
