# Supplementary material for: Study of Global Transcriptional Changes of N-GlcNAc2 Proteins-Producing T24 Bladder Carcinoma Cells under Glucose Deprivation
Source: PLoS One. 2013 Apr 1;8(4):e60397. doi: 10.1371/journal.pone.0060397 (PMC3613393; doi:10.1371/journal.pone.0060397)
Supplement: Table S3 — Lists of genes, which statically two fold up- and down-changed in most significantly up- (A) and down (B)-regulated pathways (p<0.0001) (Ref. Table 1). (PDF) [file pone.0060397.s003.pdf]

Table S3. Lists of genes, which statically two fold up- and down-changed in most significantly up- (A) and down (B)-regulated pathways ($p < 0.0001$) (Ref. Table 1).

A

Pathway										RPKM values					RPKM values (relative)				
AP1	ATF-2	RAC-1	p53	Glypican	URP	HIF-1	IGF	UDP-GlcNAc	Gene Symbol	25mM	0mM_3h	0mM_6h	0mM_9h	0mM_24h	25mM	0mM_3h	0mM_6h	0mM_9h	0mM_24h
		ARHGEF2							ARHGEF2	20.63	40.54	51.00	58.22	56.39	1.00	1.97	2.47	2.82	2.73
					ASNS				ASNS	9.76	62.31	119.11	110.96	90.32	1.00	6.38	12.20	11.37	9.25
	ATF3	ATF3	ATF3		ATF3				ATF3	0.43	17.05	9.28	9.93	15.38	1.00	39.65	21.58	23.09	35.76
ATF4		ATF4		ATF4	ATF4				ATF4	98.82	274.43	283.24	336.25	613.16	1.00	2.78	2.87	3.40	6.20
			BCL2A1						BCL2A1	3.17	20.73	21.44	10.19	0.77	1.00	6.54	6.76	3.21	0.24
CASP3									CASP3	16.55	28.61	52.42	39.91	8.40	1.00	1.73	3.17	2.41	0.51
				CTGF					CTGF	12.11	253.60	174.48	146.08	32.50	1.00	20.95	14.41	12.07	2.68
				CYR61					CYR61	49.48	499.28	254.48	194.17	87.82	1.00	10.09	5.14	3.92	1.77
	DDIT3	DDIT3		DDIT3					DDIT3	19.19	408.29	393.41	512.04	490.88	1.00	21.28	20.50	26.68	25.58
		DNAJC3				DNAJC3			DNAJC3	13.76	27.24	81.02	94.22	34.61	1.00	1.98	5.89	6.85	2.52
	DUSP1	DUSP1	DUSP1	DUSP1					DUSP1	34.97	246.35	142.87	108.02	64.10	1.00	7.04	4.09	3.09	1.83
	DUSP5	DUSP5	DUSP5						DUSP5	14.67	74.60	72.74	69.99	111.20	1.00	5.09	4.96	4.77	7.58
						EDN1			EDN1	0.19	4.75	2.82	1.02	0.42	1.00	24.61	14.59	5.26	2.18
EGR2									EGR2	0.05	0.39	0.20	0.26	0.15	1.00	7.32	3.66	4.88	2.81
					EIF2AK3				EIF2AK3	4.80	8.59	11.98	11.03	6.06	1.00	1.79	2.49	2.30	1.26
				FGF2					FGF2	12.00	44.76	76.11	63.08	31.44	1.00	3.73	6.34	5.26	2.62
				FHL2					FHL2	27.10	99.41	95.45	62.11	37.99	1.00	3.67	3.52	2.29	1.40
FOS		FOS							FOS	0.57	3.94	4.15	5.52	9.01	1.00	6.91	7.27	9.68	15.80
	GADD45A	GADD45A	GADD45A						GADD45A	25.00	269.26	240.30	188.47	142.88	1.00	10.77	9.61	7.54	5.72
				GADD45B					GADD45B	6.74	46.84	26.59	24.79	25.17	1.00	6.95	3.95	3.68	3.73
								GFPT1	GFPT1	15.18	26.74	92.26	93.17	53.88	1.00	1.76	6.08	6.14	3.55
					HERPUD1				HERPUD1	23.87	289.16	304.54	289.10	191.89	1.00	12.11	12.76	12.11	8.04
HMOX1						HMOX1			HMOX1	34.71	295.68	340.13	548.89	691.14	1.00	8.52	9.80	15.81	19.91
			HSPA1A						HSPA1A	12.61	38.75	70.29	107.30	217.86	1.00	3.07	5.57	8.51	17.28
				ID2		ID2			ID2	1.34	4.40	2.92	3.24	2.67	1.00	3.28	2.18	2.41	1.99
					IGFBP1	IGFBP1	IGFBP1		IGFBP1	0.62	3.07	3.81	2.09	8.44	1.00	4.92	6.12	3.35	13.55
			IGFBP3				IGFBP3		IGFBP3	1.38	6.00	6.44	5.58	6.53	1.00	4.34	4.67	4.04	4.73
							IGFBP5		IGFBP5	0.04	0.29	0.17	0.21	0.01	1.00	6.58	3.81	4.85	0.23
	IL23A	IL23A							IL23A	0.24	1.13	1.28	1.11	0.76	1.00	4.64	5.24	4.56	3.12
IL6	IL6	IL6							IL6	5.76	104.77	99.39	59.55	33.87	1.00	18.19	17.26	10.34	5.88
IL8	IL8			IL8					IL8	127.06	1109.16	1007.69	684.11	173.11	1.00	8.73	7.93	5.38	1.36
	JDP2	JDP2							JDP2	1.80	5.49	5.68	7.27	10.52	1.00	3.05	3.15	4.04	5.84
				KLF4					KLF4	2.43	6.61	7.71	9.02	17.33	1.00	2.72	3.17	3.71	7.13
			MDM4						MDM4	3.70	6.49	8.96	7.13	3.72	1.00	1.75	2.42	1.93	1.01
MMP1							MMP1		MMP1	9.25	25.52	53.35	49.78	14.98	1.00	2.76	5.77	5.38	1.62
			NLRC4						NLRC4	0.19	0.21	0.81	1.20	3.21	1.00	1.06	4.16	6.18	16.58
				NRG1					NRG1	2.11	8.65	9.42	6.41	3.80	1.00	4.10	4.46	3.04	1.80
				PAWR					PAWR	17.80	30.59	48.69	42.10	28.42	1.00	1.72	2.74	2.37	1.60
								PGM3	PGM3	19.93	21.09	66.12	64.49	19.97	1.00	1.06	3.32	3.24	1.00
		PIP5K1B		PIP5K1B					PIP5K1B	0.00	0.05	0.05	0.06	0.08	nd	nd	nd	nd	nd
PLAUR									PLAUR	17.91	35.14	40.01	53.27	23.69	1.00	1.96	2.23	2.97	1.32
			PLK3						PLK3	6.77	34.62	21.50	21.97	21.85	1.00	5.11	3.18	3.25	3.23
PRKCE									PRKCE	3.05	3.91	7.67	9.36	8.05	1.00	1.28	2.51	3.07	2.64
		RASGRF2							RASGRF2	0.12	0.12	0.69	1.28	1.77	1.00	1.00	5.71	10.67	14.71
RCAN1				RCAN1					RCAN1	6.96	20.96	26.26	23.58	8.91	1.00	3.01	3.77	3.39	1.28
			RCHY1						RCHY1	4.00	4.89	10.05	9.56	4.71	1.00	1.22	2.51	2.39	1.18
	SERPINB5	SERPINB5	SERPINB5						SERPINB5	20.43	53.00	70.33	39.28	19.29	1.00	2.59	3.44	1.92	0.94
			SERPINE1			SERPINE1			SERPINE1	73.79	394.05	202.27	155.38	29.30	1.00	5.34	2.74	2.11	0.40

				SGK1							SGK1	2.09	37.07	28.06	18.30	8.75	1.00	17.74	13.42	8.76	4.19
SLC3A2											SLC3A2	64.37	222.40	245.56	380.13	1580.55	1.00	3.46	3.81	5.91	24.55
				SMURF2							SMURF2	42.25	101.49	139.90	127.23	31.84	1.00	2.40	3.31	3.01	0.75
	TGFB2	TGFB2									TGFB2	0.17	1.98	1.22	0.51	0.97	1.00	11.85	7.29	3.06	5.82
				TGIF1							TGIF1	22.73	35.96	61.43	54.59	54.73	1.00	1.58	2.70	2.40	2.41
THBD											THBD	2.02	9.86	37.12	28.75	13.05	1.00	4.88	18.37	14.23	6.46
						TXNRD1					TXNRD1	123.12	238.09	314.73	406.64	582.04	1.00	1.93	2.56	3.30	4.73
								UAP1			UAP1	50.35	165.14	325.21	292.21	61.24	1.00	3.28	6.46	5.80	1.22
						VEGFA					VEGFA	27.05	50.26	73.04	91.09	80.11	1.00	1.86	2.70	3.37	2.96
					WIPI1						WIPI1	17.03	13.26	45.87	78.72	62.05	1.00	0.78	2.69	4.62	3.64
					XBP1						XBP1	54.63	200.41	168.41	134.42	173.54	1.00	3.67	3.08	2.46	3.18

B

				**Pathway**								RPKM values					RPKM values (relative)				
EPHB	Fatty acid	Androgen	Glypican	Syndecan	beta catenin	ncWnt	Wnt	N-cadherin	PLK1	G2/M transition	Gene Symbol	**25mM**	**0mM_3h**	**0mM_6h**	**0mM_9h**	**0mM_24h**	**25mM**	**0mM_3h**	**0mM_6h**	**0mM_9h**	**0mM_24h**
	ACACA										ACACA	25.54	18.55	11.81	8.19	16.44	1.00	0.73	0.46	0.32	0.64
	ACACB										ACACB	0.88	0.76	0.38	0.34	0.31	1.00	0.86	0.43	0.39	0.35
	AGPAT3										AGPAT3	14.86	10.36	5.98	6.28	11.43	1.00	0.70	0.40	0.42	0.77
	AGPAT4										AGPAT4	2.81	1.89	1.06	1.48	1.24	1.00	0.67	0.38	0.53	0.44
			BCL9	BCL9	BCL9	BCL9	BCL9	BCL9			BCL9	3.95	1.10	1.02	2.07	2.94	1.00	0.28	0.26	0.52	0.74
									BUB1B		BUB1B	26.19	16.76	9.89	8.89	1.80	1.00	0.64	0.38	0.34	0.07
								CAMK2G			CAMK2G	20.64	12.67	8.07	9.82	16.51	1.00	0.61	0.39	0.48	0.80
									CDC14B		CDC14B	4.62	3.33	1.85	1.51	3.23	1.00	0.72	0.40	0.33	0.70
			CDC25B						CDC25B	CDC25B	CDC25B	112.41	80.97	35.67	33.92	34.95	1.00	0.72	0.32	0.30	0.31
										CEP250	CEP250	16.69	10.09	4.83	6.98	11.84	1.00	0.60	0.29	0.42	0.71
				CHD7		CHD7					CHD7	5.09	1.80	1.01	1.44	2.59	1.00	0.35	0.20	0.28	0.51
		CTDSP1	CTDSP1	CTDSP1	CTDSP1	CTDSP1	CTDSP1	CTDSP1			CTDSP1	34.71	17.73	10.27	14.15	30.63	1.00	0.51	0.30	0.41	0.88
		CTDSP2	CTDSP2	CTDSP2	CTDSP2	CTDSP2	CTDSP2	CTDSP2			CTDSP2	45.01	19.13	12.71	16.87	31.69	1.00	0.42	0.28	0.37	0.70
	DGAT2										DGAT2	2.14	0.73	0.29	0.22	1.27	1.00	0.34	0.13	0.10	0.59
									DLGAP5		DLGAP5	41.36	29.86	19.29	13.88	1.51	1.00	0.72	0.47	0.34	0.04
DNM1											DNM1	6.64	4.89	3.16	2.86	1.75	1.00	0.74	0.47	0.43	0.26
			EEF2K								EEF2K	9.32	5.79	2.91	3.84	6.31	1.00	0.62	0.31	0.41	0.68
EFNA5											EFNA5	6.07	2.91	1.81	2.37	8.30	1.00	0.48	0.30	0.39	1.37
		EHMT2									EHMT2	20.74	14.31	7.16	8.70	23.06	1.00	0.69	0.35	0.42	1.11
	ELOVL3										ELOVL3	0.58	0.14	0.02	0.06	0.06	1.00	0.23	0.03	0.10	0.10
	ELOVL6										ELOVL6	17.46	7.23	4.10	4.98	5.92	1.00	0.41	0.23	0.28	0.34
EPHB4											EPHB4	12.38	5.34	1.47	2.49	10.24	1.00	0.43	0.12	0.20	0.83
									ERC1		ERC1	19.62	10.51	5.18	8.45	11.47	1.00	0.54	0.26	0.43	0.58
			ETV1								ETV1	6.66	2.86	0.78	0.30	0.46	1.00	0.43	0.12	0.05	0.07
			FAM123B	FAM123B		FAM123B	FAM123B				FAM123B	1.39	0.48	0.38	0.75	1.30	1.00	0.35	0.27	0.54	0.94
		FOXO1									FOXO1	4.80	3.57	1.08	0.85	2.07	1.00	0.74	0.22	0.18	0.43
			FZD1				FZD1				FZD1	0.65	0.38	0.22	0.22	0.48	1.00	0.58	0.33	0.34	0.74
			FZD5				FZD5				FZD5	1.81	0.80	0.61	0.98	2.13	1.00	0.44	0.34	0.54	1.18
			FZD7	FZD7			FZD7				FZD7	1.80	0.79	0.54	0.78	1.58	1.00	0.44	0.30	0.43	0.88
			FZD8				FZD8				FZD8	17.95	5.02	3.19	3.45	6.85	1.00	0.28	0.18	0.19	0.38
								GAB1			GAB1	2.81	0.84	0.47	0.50	1.46	1.00	0.30	0.17	0.18	0.52
GRB7											GRB7	2.40	0.20	0.06	0.15	0.41	1.00	0.08	0.02	0.06	0.17
		HIP1	HIP1	HIP1	HIP1	HIP1	HIP1	HIP1			HIP1	10.17	5.90	2.38	2.23	2.30	1.00	0.58	0.23	0.22	0.23
		HOXB13									HOXB13	5.57	1.24	0.62	0.49	2.56	1.00	0.22	0.11	0.09	0.46
								IL17RD			IL17RD	1.68	0.64	0.17	0.21	0.45	1.00	0.38	0.10	0.12	0.27
			INCENP	INCENP	INCENP	INCENP	INCENP	INCENP	INCENP		INCENP	26.85	13.05	9.76	13.60	5.45	1.00	0.49	0.36	0.51	0.20
KALRN											KALRN	0.42	0.40	0.19	0.12	0.07	1.00	0.95	0.45	0.29	0.17
		KDM3A	KDM3A	KDM3A	KDM3A	KDM3A	KDM3A	KDM3A			KDM3A	21.78	6.01	7.50	10.04	17.27	1.00	0.28	0.34	0.46	0.79

									KIF20A		KIF20A	0.42	0.40	0.19	0.12	0.07	1.00	0.95	0.45	0.29	0.17
			KREMEN1				KREMEN1				KREMEN1	3.32	1.57	0.87	0.78	1.77	1.00	0.47	0.26	0.23	0.53
				LAMA3							LAMA3	2.31	1.19	0.82	0.82	0.52	1.00	0.51	0.35	0.35	0.22
	LPIN1										LPIN1	30.03	20.26	10.12	8.85	19.28	1.00	0.67	0.34	0.29	0.64
		MAP2K6	MAP2K6								MAP2K6	1.63	1.19	0.84	0.60	2.50	1.00	0.73	0.52	0.37	1.53
			MAP3K12								MAP3K12	10.36	5.65	3.08	4.87	10.05	1.00	0.55	0.30	0.47	0.97
			MAP3K4								MAP3K4	15.76	13.31	6.59	5.54	5.19	1.00	0.84	0.42	0.35	0.33
			MAP3K8								MAP3K8	1.52	0.95	0.34	0.41	0.55	1.00	0.63	0.22	0.27	0.36
		MAPK14	MAPK14						MAPK14		MAPK14	23.44	11.99	9.13	9.29	10.80	1.00	0.51	0.39	0.40	0.46
			MAPKAPK3								MAPKAPK3	14.94	8.93	5.36	6.75	39.82	1.00	0.60	0.36	0.45	2.66
									MXI1		MXI1	32.60	12.19	8.15	7.40	24.02	1.00	0.37	0.25	0.23	0.74
		NCOA1									NCOA1	7.26	5.33	3.09	3.46	7.27	1.00	0.73	0.43	0.48	1.00
		NCOA2	NCOA2	NCOA2	NCOA2	NCOA2	NCOA2	NCOA2			NCOA2	7.60	3.44	1.98	3.09	5.16	1.00	0.45	0.26	0.41	0.68
									NFKBIA		NFKBIA	127.34	177.51	59.73	35.89	32.56	1.00	1.39	0.47	0.28	0.26
		NRIP1	NRIP1	NRIP1	NRIP1	NRIP1	NRIP1	NRIP1			NRIP1	21.42	16.07	11.40	7.13	3.90	1.00	0.75	0.53	0.33	0.18
										NUMA1	NUMA1	57.90	31.13	18.94	25.99	35.30	1.00	0.54	0.33	0.45	0.61
					PAG1			PAG1			PAG1	1.24	0.38	0.18	0.18	0.29	1.00	0.30	0.15	0.14	0.23
PAK1			PAK1	PAK1		PAK1			PAK1		PAK1	36.44	23.38	16.18	11.67	20.02	1.00	0.64	0.44	0.32	0.55
		PATZ1	PATZ1	PATZ1	PATZ1	PATZ1	PATZ1	PATZ1			PATZ1	14.57	8.47	4.54	6.85	21.29	1.00	0.58	0.31	0.47	1.46
		POU2F1									POU2F1	3.31	1.55	1.07	1.62	2.83	1.00	0.47	0.32	0.49	0.85
	PPARA										PPARA	7.09	2.38	1.73	3.60	6.88	1.00	0.34	0.24	0.51	0.97
				PPARG		PPARG					PPARG	66.87	20.14	19.18	14.61	34.35	1.00	0.30	0.29	0.22	0.51
				PRKCD							PRKCD	11.77	5.91	3.30	6.31	13.90	1.00	0.50	0.28	0.54	1.18
			PTCH1								PTCH1	1.76	0.38	0.26	0.45	1.78	1.00	0.21	0.15	0.25	1.01
										RASGRF1	RASGRF1	0.33	0.16	0.10	0.11	0.64	1.00	0.47	0.29	0.32	1.92
								RIN2			RIN2	47.36	16.61	16.32	16.05	7.19	1.00	0.35	0.34	0.34	0.15
								RPS6KA1			RPS6KA1	29.76	17.04	4.51	5.93	23.78	1.00	0.57	0.15	0.20	0.80
								RUNX2			RUNX2	6.16	1.67	1.45	0.98	0.97	1.00	0.27	0.23	0.16	0.16
	RXRA										RXRA	13.98	7.25	2.54	3.86	11.05	1.00	0.52	0.18	0.28	0.79
			SALL4	SALL4	SALL4	SALL4	SALL4	SALL4			SALL4	0.57	0.41	0.18	0.16	0.49	1.00	0.71	0.31	0.27	0.85
			SMAD3								SMAD3	69.01	71.86	30.38	20.14	10.15	1.00	1.04	0.44	0.29	0.15
			SMARCA4	SMARCA4	SMARCA4	SMARCA4	SMARCA4	SMARCA4			SMARCA4	31.26	23.05	11.86	14.09	29.51	1.00	0.74	0.38	0.45	0.94
			SOS1					SOS1			SOS1	13.94	6.88	4.13	4.63	7.28	1.00	0.49	0.30	0.33	0.52
SRC		SRC						SRC	SRC		SRC	21.64	11.10	5.92	9.55	27.57	1.00	0.51	0.27	0.44	1.27
		SVIL	SVIL	SVIL	SVIL	SVIL	SVIL	SVIL			SVIL	3.05	2.58	1.23	0.94	1.21	1.00	0.85	0.40	0.31	0.40
	TBL1XR1										TBL1XR1	37.46	24.16	13.33	8.00	5.50	1.00	0.64	0.36	0.21	0.15
		TCF4	TCF4	TCF4	TCF4	TCF4	TCF4	TCF4			TCF4	4.81	3.57	1.86	0.95	1.04	1.00	0.74	0.39	0.20	0.22
			TCF7L1								TCF7L1	2.23	1.53	0.37	0.49	1.21	1.00	0.69	0.17	0.22	0.54
			TCF7L2	TCF7L2	TCF7L2	TCF7L2	TCF7L2	TCF7L2			TCF7L2	14.75	6.97	5.23	5.58	8.74	1.00	0.47	0.35	0.38	0.59
									TDRD7		TDRD7	12.74	7.55	4.46	5.35	8.79	1.00	0.59	0.35	0.42	0.69
			TLE1	TLE1	TLE1	TLE1	TLE1	TLE1			TLE1	8.62	5.63	2.12	2.22	6.65	1.00	0.65	0.25	0.26	0.77
			TLE4	TLE4	TLE4	TLE4	TLE4	TLE4			TLE4	9.70	5.10	2.67	2.33	3.03	1.00	0.53	0.27	0.24	0.31
									WEE1	WEE1	WEE1	15.54	9.73	7.71	6.45	3.44	1.00	0.63	0.50	0.42	0.22
			WNT3				WNT3				WNT3	0.97	0.63	0.25	0.15	1.57	1.00	0.64	0.26	0.15	1.61
								ZBTB33			ZBTB33	13.69	7.71	6.06	5.48	8.87	1.00	0.56	0.44	0.40	0.65
		ZNF318	ZNF318	ZNF318	ZNF318	ZNF318	ZNF318	ZNF318			ZNF318	5.69	2.98	1.75	2.35	4.65	1.00	0.52	0.31	0.41	0.82
