# Supplementary material for: Correction: Study of Global Transcriptional Changes of N-GlcNAc2 Proteins-Producing T24 Bladder Carcinoma Cells under Glucose Deprivation
Source: PLoS One. 2013 Nov 12;8(11):10.1371/annotation/053f227a-3847-41e2-a8c4-a5530a282a29. doi: 10.1371/annotation/053f227a-3847-41e2-a8c4-a5530a282a29 (PMC3825713; doi:10.1371/annotation/053f227a-3847-41e2-a8c4-a5530a282a29)
Supplement: Supplementary file 1 [file pone.053f227a-3847-41e2-a8c4-a5530a282a29.s001.doc]

**Table S1. Oligonucleotides used for qRT-PCR**

| Gene | Forward | Reverse |
| --- | --- | --- |
| *AURKA*  *AURKB* | ATTTCAGGACCTGTTAAGCTAC  CACTTCACAATTGATGACTTTGAG | TCTGATTCTGAACCGGCTTGTG  CAGGATGTTGGGATGGTGCAG |
| *CDK1*  *NEK2* | TGGAGTTGTATAAGGGTAGAC  CCCTGAAGGAATGCCACAGAC | ATAAGCACATCCTGAAGACTGAC  TGCGATTCATTTGTTCAGGAGAC |
| *PLK1* | AGATCAACTTCTTCCAGGATCAC | TGAGACGGTTGCTGGCCGAG |
| *ATF3*  *ATF4*  *FOXO1*  *GAPDH* | TGCCTGTCCCCTCCTGGGTC  TCCAACAACAGCAAGGAGGATG  AAGCTCCCAAGTGACTTGGATG  GGGAGCCAAAAGGGTCATCATC | TCTTCTTCAGGGGCTACCTCG  GGGCAAAGAGATCACAAGTGTC  AGTACTTTTAAGTGTAACCTGCTC  TGGCATGGACTGTGGTCATGAG |
